# Coupled ocean-atmosphere dynamics of the 2017 extreme coastal El Niño

**DOI:** 10.1038/s41467-018-08258-8

**Published:** 2019-01-17

**Authors:** Qihua Peng, Shang-Ping Xie, Dongxiao Wang, Xiao-Tong Zheng, Hong Zhang

**Affiliations:** 1 State Key Laboratory of Tropical Oceanography, South China Sea Institute of Oceanology, Chinese Academy of Sciences, Guangzhou, 510301 China; 2 Scripps Institution of Oceanography, University of California San Diego, La Jolla, CA 92093 USA; 3 University of Chinese Academy of Sciences, Beijing, 100049 China; 4 Physical Oceanography Laboratory, Ocean University of China, and Qingdao National Laboratory for Marine Science and Technology, 266100 Qingdao, China; 5 Joint Institute for Regional Earth System Science and Engineering, University of California, Los Angeles, 90095 CA USA

## Abstract

In March 2017, sea surface temperatures off Peru rose above 28 °C, causing torrential rains that affected the lives of millions of people. This coastal warming is highly unusual in that it took place with a weak La Niña state. Observations and ocean model experiments show that the downwelling Kelvin waves caused by strong westerly wind events over the equatorial Pacific, together with anomalous northerly coastal winds, are important. Atmospheric model experiments further show the anomalous coastal winds are forced by the coastal warming. Taken together, these results indicate a positive feedback off Peru between the coastal warming, atmospheric deep convection, and the coastal winds. These coupled processes provide predictability. Indeed, initialized on as early as 1 February 2017, seasonal prediction models captured the extreme rainfall event. Climate model projections indicate that the frequency of extreme coastal El Niño will increase under global warming.

## Abstract (web summary)

The extreme coastal El Niño of March 2017 caused devastating flooding in coastal Peru but its mechanism remains unclear. Here the authors investigate the physical processes using observations and model simulations and suggest that such extreme coastal flooding is predictable and will become more frequent as climate warms.

## Introduction

**Fig. 1:**
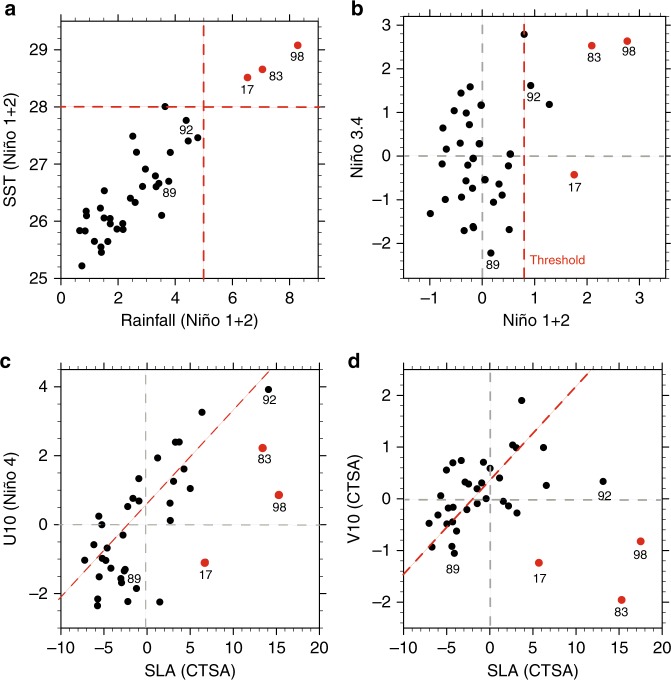
Coastal El Niño-related indices over the satellite era. Scatter diagram for **a** March Niño 1 + 2 rainfall (mm day^−^^1^) and SST (°C); **b** FM Niño 1+2 SSTAs and NDJ Niño 3.4 index (°C), red dashed line indicates convective threshold (27 °C in current climate); **c** FMA sea level anomalies (SLA; cm) over the coastal region of tropical South America (CTSA; 85 °W–80 °W, 10 °S–0°) and zonal wind anomalies (m s^−1^) over Niño 4 region; **d** JFMA SLA (cm) and meridional wind anomalies (m s^−^^1^) over CTSA

**Fig. 2:**
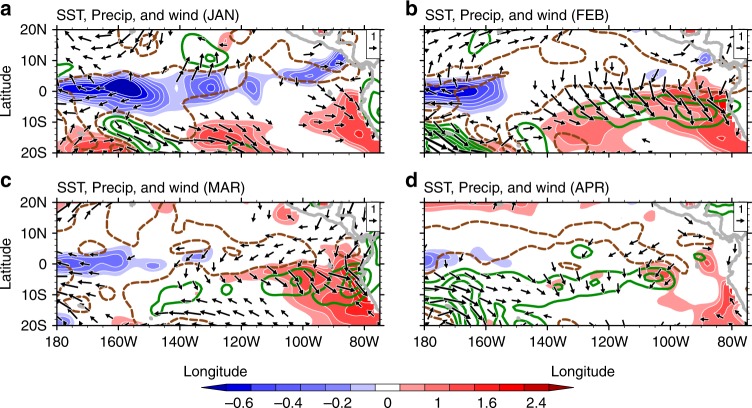
Climate conditions of JFMA 2017. Anomalous SST (°C; color shading), precipitation (contours at intervals of 2 mm day^−1^; negative in brown and positive in green), and surface wind velocity (m s^−1^; vectors) for **a** January, **b** February, **c** March, and **d** April

Unlike lush Central America north of the equator, the Pacific coast of Peru and Ecuador is kept cool and dry by intense upwelling of cold water from beneath. Even in March when sea surface temperature (SST) reaches the annual maximum in the Southern Hemisphere, there is little rainfall along the coastal Peru because SSTs remain below the convective threshold (~27 °C in current climate^1^). Only strong warm events cause deep convection and heavy rainfall in this region. During January–March 2017, torrential rains (~6 mm day^−1^; Fig. 1a) devastated northern Peru, causing extreme flooding and widespread landslides that resulted in at least 200 deaths and a huge loss of properties^2^. This is one of the worst floods of Peru on record in terms of both the rainfall amount and the number of people affected. Extremely high SSTs in excess of 28 °C were observed in the coastal regions (Fig. 1a), promoting atmospheric deep convection. Similar events of extreme rainfall and coastal ocean warming took place in 1983 and 1998 (Fig. 1a, b) but they were each associated with a strong basin-wide El Niño state. The strong coastal El Niño of 2017 was highly unusual in that it was confined to the coastal region and preceded by a weak basin-scale La Niña (Figs. 1b and 2).

El Niño originally referred to an abnormal warm coastal event off Peru and Ecuador. It is not until the 1960s that scientists realized that El Niño is not limited to the coast, but part of the basin-scale warming involving strong ocean–atmosphere interactions^3–6^. Now it is widely recognized that the strong eastern-Pacific warming is mainly caused by westerly wind anomalies in the central Pacific through equatorial wave adjustments. In this classic view, strong coastal warming is associated with a basin-scale El Niño (or eastern-Pacific El Niño) (Fig. 1b) (e.g., in 1983, 1987, 1992, and 1998). However, the relation between coastal warming and basin-scale El Niño is not always strong. Weak coastal warming sometimes occurs with a strong negative Niño 3.4 index during a central-Pacific La Niña (or La Niña-Modoki)^7–10^ (Fig. 1b). Typically a central-Pacific La Niña is characterized by strong negative Niño 4 SSTAs and weak positive Niño 1 + 2 SSTAs with small impacts on rainfall off Peru and Ecuador. In February–April (FMA) 2017, however, weak negative SSTAs were observed in the central Pacific with extreme coastal warming and a dramatic increase in rainfall off Peru. The 2017 case differs from the eastern-Pacific El Niño or the central-Pacific La Niña in spatial pattern, cause, and impact, so we define the 2017 case as an extreme coastal El Niño (see Methods). Similar extreme coastal El Niño events occurred in 1891 and 1925 (refs. ^2,11^).

While basin-scale El Niño (of both eastern and central-Pacific types) has been studied extensively^12–16^, mechanisms for extreme coastal El Niño are not well understood. The 2017 coastal warming captured the immediate attention of the scientific community. Several explanations—some mutually conflicting—have been proposed, ranging from anomalous northerly coastal winds^2,11^ to westerly wind anomalies in the eastern equatorial Pacific^17^. In March 2017, a large-scale meridional dipole pattern of rainfall anomalies was observed across the eastern Pacific with anomalous northerly winds. Wind-evaporation-SST (WES) feedback^18^ is important for this eastern Pacific Intertropical Convergence Zone (ITCZ) dipole (EPID) mode^19^. In studying a coastal event of 1925, Takahashi and Martínez^11^ suggested a possibility that concurrent La Niña conditions in the central Pacific might be in favor of atmospheric convection off Peru by reducing atmospheric stability. These hypotheses have not yet been rigorously tested from observations together with realistic models. Specifically, important questions remain unanswered: what is the relative importance of remote and local forcing in the 2017 extreme coastal El Niño? What is the role of local air–sea interaction? Is this extreme event predictable? Why is the extreme coastal El Niño so rare? How will it change in the face of increasing greenhouse warming?

The present study investigates these questions using a wide range of ocean–atmospheric observations, both in situ and from space. We further take advantage of comprehensive oceanic and atmospheric general circulation models that show remarkable skills in simulating the extreme event of 2017. Experiments with an oceanic general circulation model (OGCM) show that both the remotely forced downwelling Kelvin waves and coastal wind anomalies contribute to the extreme coastal El Niño. Atmospheric general circulation model (AGCM) experiments reveal a previously unknown ocean–atmosphere coupling in the coastal region, between coastal warming, atmospheric deep convection, and alongshore winds. The coastal coupling implies predictability at monthly leads, which we confirm from operational seasonal forecast.

## Results

### Oceanic dynamic processes

**Fig. 3:**
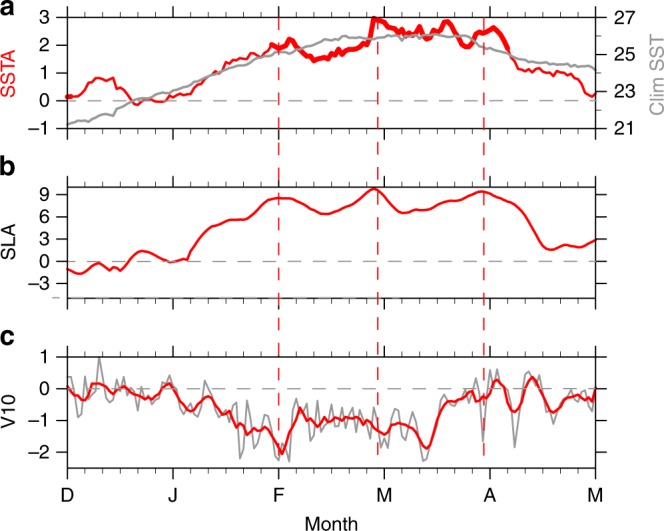
Evolution of the 2017 extreme coastal El Niño. Time series of **a** climatological SST (°C; gray line) and SSTA (°C; red line), the bold red line indicates SSTs above 27 °C; **b** SLA (cm) and **c** meridional alongshore wind anomalies (m s^−^1; gray line is obtained from raw data and red line indicates 5-day running mean result). SSTs and winds are averaged in CTSA while SLA is averaged in 85 °W–95 °W, 2° S–2 °N to measure equatorial waves in the eastern Pacific

In late 2016, a La Niña with moderate amplitude occurred over the tropical Pacific Ocean with near neutral SSTAs off Peru and persistent weak SST warming along the west coast of subtropical South America. SSTAs over the coastal region of tropical South America (CTSA; 85 °W–80 °W, 10 °S–0°) started to increase in mid-January 2017 and reached +3 °C in March while weak negative SSTAs were observed over Niño 3 and Niño 4 regions (Figs. 2 and 3a). Extreme SSTAs at some coastal stations of Peru exceeded +5 °C (Supplementary Table 1). The strong SST warming persisted into April. In detail, the SST warming came in four episodes around January 30, February 28, March 18, and March 28, respectively, and then decayed rapidly. SST returned to normal by the end of April (Fig. 3a).

As a first step, we examine a mixed layer heat budget to assess the processes that control the SST evolution in the CTSA. The results (Supplementary Figure 1a) suggest that the subsurface term dominates the mixed layer warming throughout the period, with weakened upwelling south of the equator (Supplementary Figure 1c). Southward advection associated with the intensified coastal flow played a secondary role.

The subsurface term can be due to both the alongshore wind anomalies and the downwelling Kelvin waves through changes in upwelling and coastal stratification. In the next section, we use the MIT General Circulation Model (MITgcm) to examine the underlying mechanisms for the 2017 extreme coastal El Niño.

### The role of equatorial Kelvin waves

**Fig. 4:**
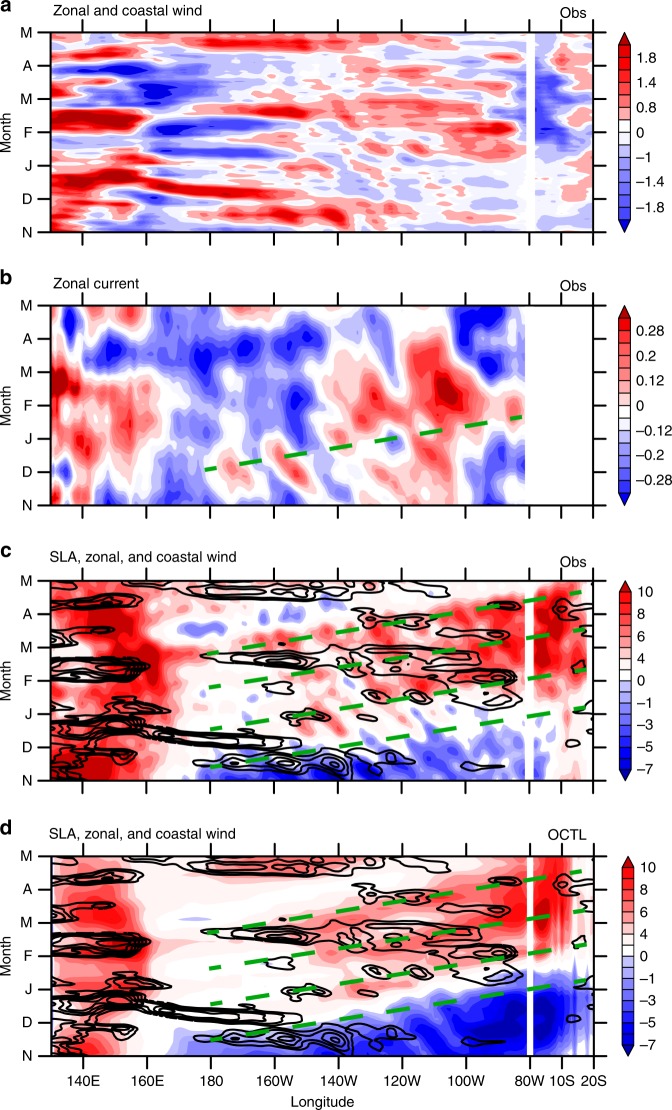
Longitude–time evolution of the 2017 extreme coastal El Niño. Hovmöller diagrams for: **a** zonal and coastal wind anomalies (color shading; m s^−^^1^; negative values along the coast of South America indicates northerly wind anomalies); **b** zonal current (color shading; m s^−1^), green dashed line indicates Kelvin wave propagation; **c** SLA (color shading; cm) and zonal wind anomalies (contours with an interval of 0.5 m s^−^^1^; positive solid and negative omitted) from observations; **d** same as **c** but from MITgcm OCTL. All meridionally averaged in 2 °S–2 °N along the equator and zonally averaged in 80 °W–85 °W along the coast

Hovmöller diagrams of sea level anomalies (SLA) and zonal currents along the equator and west coast of South America indicate that marked downwelling Kelvin waves propagated from 160 °W towards the American coast with a phase speed of 2.73 m s^−1^ during the boreal spring of 2017 (Fig. 4b, c). Upon arriving at the coast, the equatorial Kelvin waves propagated southward along the Peruvian coast as coastal Kelvin waves. The SLA of the downwelling Kelvin waves amounted to +10 cm along the coast (Fig. 3b and Supplementary Table 1), depressing the thermocline (Supplementary Figure 1c and in situ observations, Figs. 3.2.5.1 and 3.2.5.2 of ref. ^20^) and raising SST there. These positive SLAs lasted for about 3 months. Note that the intraseasonal SLA episodes are consistent with those of SSTA (Fig. 3a, b). Specifically, SST started to warm at the time the downwelling Kelvin waves arrived at the Peruvian coast in late January with the subsequent warming pulses following SLA pulses throughout February–March (FM). Finally the coastal warming decayed rapidly upon the arrival of upwelling Kelvin waves (Fig. 4c).

**Fig. 5:**
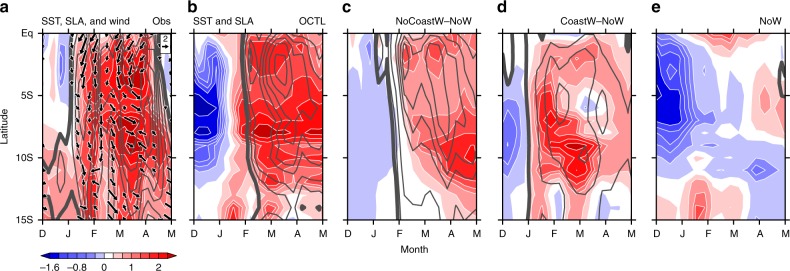
Latitude–time evolution of the 2017 extreme coastal El Niño. Hovmöller diagrams for: **a** SSTA (color shading; °C), SLA (contours with an interval of 1 cm; zero thickened and negative omitted) and wind anomalies (vectors; m s^−^^1^) from observations. **b**–**e** same as **a** but from MITgcm: OCTL, NoCoastW-NoW, CoastW-NoW, and NoW, respectively. All zonally averaged in 80 °W–85 °W

To test the hypothesis that the equatorial downwelling Kelvin waves contributed to the coastal warming, we performed three experiments with MITgcm (see Methods and Supplementary Table 2). OCTL is a hindcast for 2007–2017 forced by the full forcing. As is evident in Figs. 4d and 5b, the model reproduces many features of the observed SST evolution (Figs. 4c and 5a). For instance, the simulated coastal El Niño starts from January 2017, peaks in March, and decays in April, much as in observations. In addition, the eastward propagating Kelvin waves are reproduced well by the OGCM, including the intraseasonal pulses (Fig. 4d).

We conduct an experiment where coastal wind stress anomalies over CTSA are removed. This NoCoastW run tests the combined effects of remote dynamical forcing and local thermal forcing. To further isolate the local thermal effect, we conduct another experiment, NoW, where we keep the wind stress everywhere fixed to the monthly climatology during November 2016–May 2017. Figure 5e shows that SSTAs induced by local thermal forcing are very small during FMA 2017. Although the cooling before January 2017 is much stronger than observed, it has little impacts on the extreme coastal warming in FMA 2017. The difference of NoCoastW and NoW isolates the remote dynamical forcing effect. Without the coastal wind stress anomalies, sizable warming (1–1.5 °C) remains along the coast (Fig. 5c), demonstrating the remote forcing contribution, specifically from the equatorial Kelvin waves. In addition, the evolution of sea level rise that began in late January and continued through early April 2017 is comparable between the result of NoCoastW minus NoW (Fig. 5c) and OCTL runs (Fig. 5b), implying that the coastal sea level rise is mainly caused by remote equatorial Kelvin waves. These Kelvin waves depress the thermocline in CTSA and raise SSTs. Thus, OGCM results corroborate the importance of remote equatorial Kelvin waves in the 2017 coastal El Niño.

**Fig. 6:**
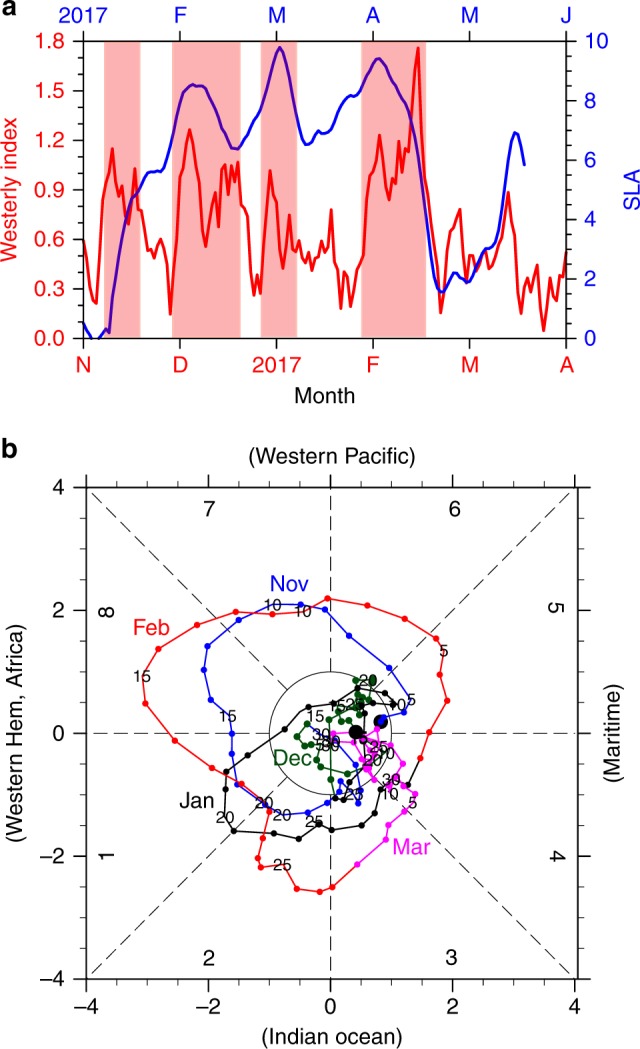
Westerly wind events and MJO activities from November 2016 to March 2017. **a** Westerly index (m s^−1^; red line; westerly wind anomalies averaged in the equatorial Pacific zonally and between 2 °S–2 °N) and SLAs (cm; blue line, averaged in 85  °W–95 °W, 2 °S–2  °N). **b** The phase–space diagram of the Wheeler and Hendon MJO indices for 1 November 2016–30 March 2017 (November in blue, December in green, January in black, February in red, and March in pink)

Westerly anomalies in the central and western equatorial Pacific excite the downwelling equatorial Kelvin waves as is clear (*r* = 0.67) from a scatter diagram for Niño 4 zonal wind anomalies and CTSA SLA (Fig. 1c). It might seem counterintuitive that in 2017 sea level rose in CTSA despite overall easterly wind anomalies in Niño 4 region (Fig. 1c). The westerly wind events (WWEs) occurred at a 2-month lead time relative to coastal SLA pulses, indicating these downwelling Kelvin waves were mainly forced by WWEs (Fig. 6a). Specifically, the first WWE in mid-November 2016 terminated the negative SLAs on the coast (Figs. 4c and 6a). The second WWE occurred in early December 2016, driving the first positive Kelvin wave pulse that arrived at the CTSA in early February 2017 (Fig. 6a). Then in early January 2017, the third WWE forced another downwelling Kelvin wave pulse, which arrived at the CTSA in early March 2017 (Fig. 6a). The last WWE took place in mid-February 2017 and excited a Kelvin wave pulse arriving at CTSA in early April (Fig. 6a). Interestingly, the MJO index was large in November 2016, late January, and February 2017, coinciding with westerly anomalies in the equatorial Pacific region^21^. Thus, these WWEs were associated with MJO events. In early December 2016 and early January 2017, there were no MJO activities, WWEs during this time were associated with a range of phenomena such as tropical cyclones or mid-latitude cold surges^22–24^.

### Coastal wind forcing

In addition to the downwelling equatorial Kelvin waves, the strong alongshore northerly wind anomalies also seem important for the growth of SST warming over CTSA through suppressing upwelling and reducing evaporation (WES feedback)^2^ (Supplementary Figure 1b). To quantify the local wind effect, we conduct an experiment named CoastW, which is forced by climatological wind stress outside CTSA while observed wind stress inside CTSA. The difference of CoastW and NoW isolates dynamical forcing of local wind stress. Figure 5d shows the local wind stress effect on coastal SST reaches 1.5 °C, comparable with the effect of the equatorial downwelling Kelvin waves.

Both the equatorial downwelling Kelvin waves and alongshore northerly anomalies are essential for the 2017 extreme coastal El Niño. While CTSA SLAs in the 2017 event are only one-third of those in 1983 and 1998 (Fig. 1c), the SSTAs of these three events are comparable. This indicates that the 2017 downwelling Kelvin waves are not strong enough to generate an extreme coastal warming, and some other process also contributes to this extreme coastal El Niño. Indeed the anomalous northerly alongshore winds in 2017 are the second largest since 1980 (Fig. 1d), working together with downwelling Kelvin waves to induce the strong coastal warming. Such a constructive interference between the equatorial Kelvin waves and coastal wind forcing is rare (Fig. 1d). For example, in January–April (JFMA) 1989, the CTSA northerly anomalies were comparable with those in 2017, but upwelling Kelvin waves prevented a strong coastal warming (Fig. 1b, d). In FM 1992, strong positive SLAs were found over CTSA, nearly twice as large as those in 2017. But weak southerly wind anomalies limited positive CTSA SSTA in FM 1992 to only half of that in 2017 (Fig. 1b). These examples further illustrate that both the downwelling Kelvin waves and local northerly alongshore wind anomalies are necessary for extreme coastal El Niño.

### SST impacts on the atmosphere

**Fig. 7:**
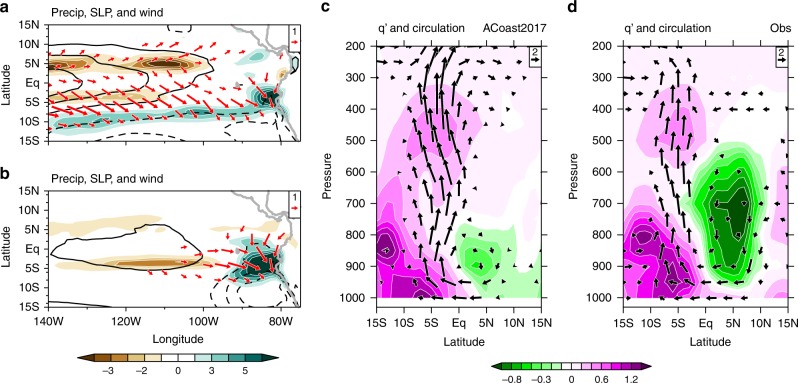
Atmospheric response to 2017 FMA SSTAs. The 10-year-mean anomalies of FMA precipitation (color shading; mm day^−^^1^), sea level pressure (contours at an interval of 10 hPa, zero omitted, and negative dashed), and surface wind anomalies (vectors; m s^−^^1^) for **a** A2017 and **b** ACoast2017. Vertical circulation anomalies (vectors; m s^−1^) and specific humidity (*q*’, g kg^−1^; color shading) along the west coast of America based on **c** ACoast2017 and **d** observations (zonally averaged in 80 °W– 85 °W)

Here we use the Community Atmosphere Model version5.3 (CAM5.3) to assess the atmospheric influence of 2017 SSTAs. The control run (ACTL) is forced by monthly climatological SSTs. We carry out an experiment (named A2017) by imposing the 2017 SSTAs. The 10-year mean precipitation and surface wind anomalies over the eastern Pacific from the A2017 run (Fig. 7a) compare well with those observed (Fig. 2). Specifically, in both observations and the A2017 run, precipitation increased over a broad band of positive SSTAs with the weakened trade winds in the southeastern tropical Pacific.

To further explore the extent to which the atmospheric response can be explained by local SSTAs over the coastal region, we perform another experiment (named ACoast2017) by imposing 2017 SSTAs only in the coastal region (90 °W–75 °W, 0–20 °S). Coastal atmospheric anomalies are reproduced. Specifically, coastal SSTAs lower sea level pressure (SLP), driving strong northerly anomalies and heavy rainfall (~7 mm day^−1^) off Peru and Ecuador (Fig. 7b). During FMA when SSTs off Peru reach the annual maximum and are close to the convective threshold, strong coastal warming can cause deep convection and heavy rainfall. In FMA 2017, anomalous upward motion off Peru extends over the entire troposphere (Fig. 7c, d), indicative of deep convection. The rainfall and wind anomalies over the coastal region are similar between the ACoast2017 and A2017 runs, demonstrating that they were mainly forced by coastal rather than basin-scale SSTAs.

### Coastal ocean–atmosphere coupling

**Fig. 8:**
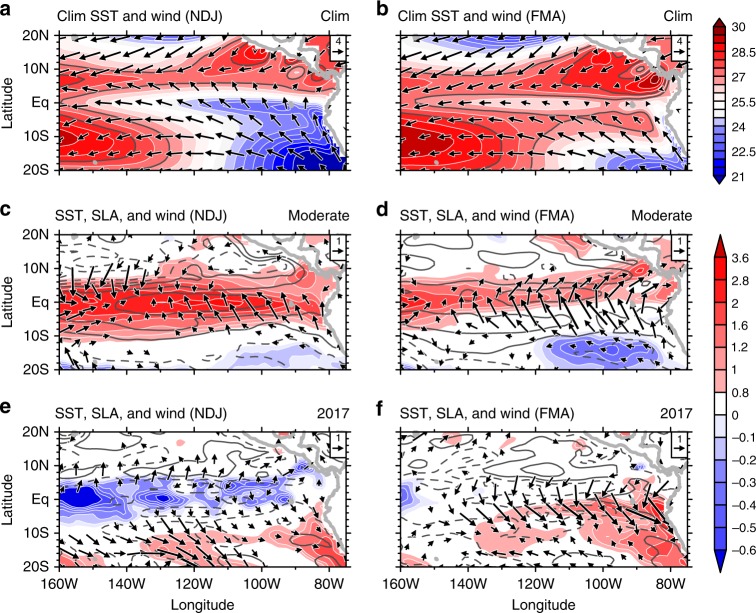
Ocean–atmospheric conditions of basin-scale moderate El Niño and the 2017 extreme coastal El Niño. Climatological SST (°C; color shading; 27 and 28 °C contours in gray line) and winds (vectors; m s^−^^1^) over the eastern Pacific for **a** NDJ and **b** FMA; composite NDJ (left) and FMA (right) SSTAs (°C; color shading), SLA (contours with an interval of 2 cm, zero omitted and negative dashed) and wind anomalies (m s^−1^; arrows) for basin-scale moderate El Niño (**c**, **d**) and the 2017 extreme coastal El Niño (**e**, **f**)

The above OGCM and AGCM experiments demonstrate strong air–sea interactions during the 2017 coastal El Niño. During November 2016–April 2017, there were northerly cross-equatorial winds in the eastern Pacific (Fig. 8e, f). These winds were not confined to the coast but extended far offshore, characterized by a basin-scale rainfall dipole pattern (Figs. 2 and 8e, f). These large-scale anomalous wind pattern can be explained by coupled dynamics associated with the EPID mode:^19^ typically, at the peak phase of a moderate El Niño (La Niña), SST anomalies are nearly symmetric about the equator (Fig. 8c). However, the climatological mean SST is strongly asymmetric over the eastern Pacific, above the convective threshold only north of the equator (Fig. 8a). As a result, the atmospheric response to symmetric SST anomalies is asymmetric about the equator. Deep convection intensified (weakened) only north of the equator, decelerating (accelerating) trade winds north of the equator and accelerating (decelerating) trade winds south of the equator (Fig. 8c, d). These anomalous trade winds drive cross-equatorial asymmetric SSTAs, inducing the EPID mode. Thus, a positive (negative) EPID mode, following a moderate El Niño (La Niña), is often characterized by southerly (northerly) wind anomalies in the southeastern tropical Pacific.

At the end of 2016, a negative EPID mode started to develop across the eastern Pacific with anomalous northerlies growing on WES feedback (Fig. 8e). In addition, there was a persistent weak warming along the west coast of subtropical South America (10 °S–30 °S) (Fig. 5a). These preexisting positive SSTAs could induce negative SLP anomalies and drive northerly anomalous winds^21^. The negative EPID mode and preexisting positive SSTAs worked together and drove anomalous northerly winds during November–January (NDJ) 2016. These northerly wind anomalies contributed to CTSA warming, setting up favorable conditions for the 2017 extreme coastal El Niño.

During November 2016–March 2017, WWEs forced strong downwelling Kelvin waves along the equator. In early February 2017, the first strong downwelling Kelvin wave pulse arrived at CTSA, depressing the thermocline and raising SSTs above the convective threshold. The resultant deep convection drove anomalous northerlies along the coast (Fig. 7c, d). In addition, the negative EPID mode peaked in FMA, forcing strong basin-scale anomalous northerly winds (Fig. 8f). Thus in FMA 2017, these two factors worked together to generate anomalous northerly winds over CTSA, which were the second largest since 1980 (Fig. 1d). These anomalous northerlies intensified coastal warming by WES feedback and the weakened upwelling (Supplementary Figures 1b and 1c). This coastal Bjerknes feedback took place during late January to early April 2017, amplifying the strong coastal warming. In late April, a weak upwelling Kelvin wave pulse terminated this positive feedback (Fig. 4c).

### Prediction of extreme coastal rainfall

**Fig. 9:**
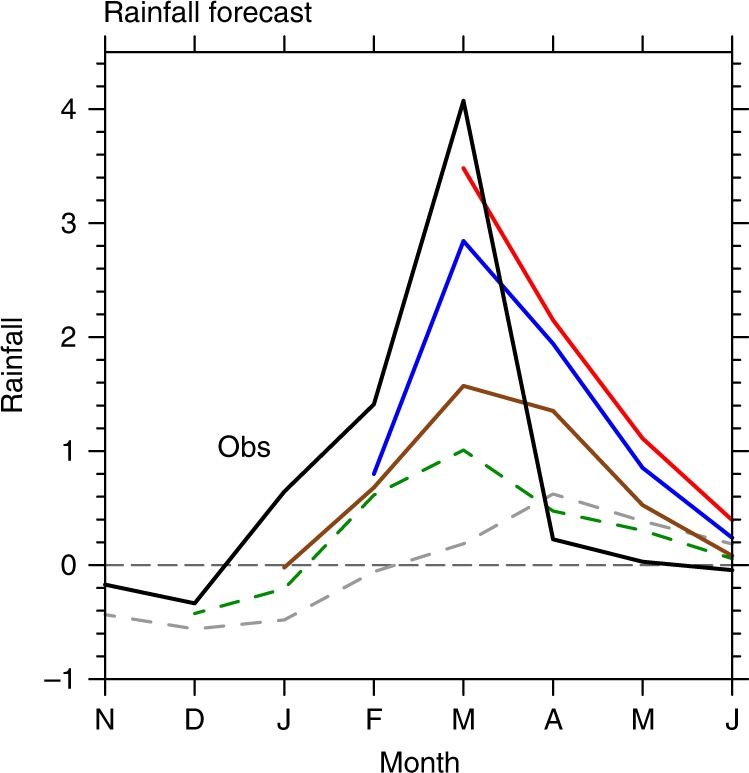
Predictions of the 2017 extreme coastal rainfall from the NMME. Rainfall (mm day^−1^) forecasts over CTSA initialized on 1 November 2016 (gray dashed), 1 December 2016 (green dashed), 1 January 2017 (brown), 1 February 2017 (blue), and 1 March 2017 (red). Also plotted in black are observations

The importance of the remotely forced Kelvin waves and resultant coastal coupling imply predictability. Here we evaluate seasonal forecast of Peruvian coastal rainfall from the North American Multi-Model Ensemble (NMME). The multimodel ensemble (MME) averages show the forecasts initialized prior to 2017 had limited skill (Fig. 9). Into 2017, many models began to forecast anomalous wet conditions in boreal spring over the CTSA region. Forecasts initialized on 1 February 2017 predicted heavy rainfall (+2.9 mm day^−1^) in mid-March. When initialized on 1 March 2017, the predicted March rainfall anomalies reached ~3.5 mm day^−1^, comparable with observations.

The initial conditions on 1 February included weak warming forced by local winds and the first pulse of downwelling Kelvin waves. Upon the arrival of the downwelling Kelvin wave pulse, CTSA SSTs exceeded the convective threshold, activating coastal Bjerknes feedback. This air–sea feedback was important to sustain or amplify the coastal warming throughout February–March 2017, explaining that rainfall prediction improves drastically with February and March initializations. SLA data are not provided in the NMME, precluding a closer look at the Kelvin waves and its relationship with SSTAs in the forecast models. The success of NMME forecasts is encouraging, allowing a lead time of up to 1 month in advance for local authorities to implement preventive measures and reduce the loss of lives and properties.

## Discussion

**Fig. 10:**
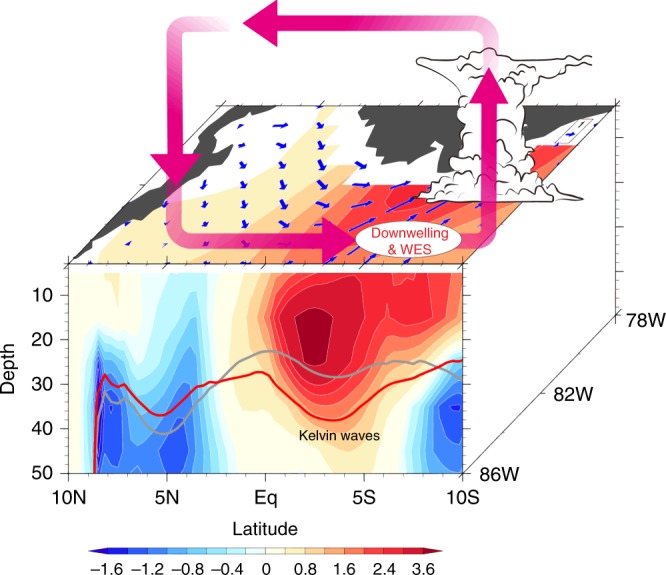
Schematic for the 2017 extreme coastal El Niño. FMA wind (m s^−^^1^; blue arrows) and temperature anomalies (°C; color shading) along the American coast, with the 20 °C isotherm for climatology and 2017 highlighted in gray and red lines, respectively. The meridional atmospheric circulation anomalies are indicated by the pink arrows. The ellipse indicates WES feedback and oceanic downwelling favorable process. The cumulonimbus south of the equator denotes deep convection

Our oceanic and atmospheric model experiments show that the combined effect of local winds and equatorial Kelvin waves caused the 2017 extreme coastal El Niño, amplified by a positive coastal Bjerknes feedback. Figure 10 is a schematic of oceanic and atmospheric processes that caused the 2017 extreme coastal El Niño. At the beginning of 2017, a negative EPID mode developed in the eastern tropical Pacific, featuring northwesterly anomalies south of the equator (Fig. 2a). In addition, preexisting SST warming along the west coast of subtropical South America also favored anomalous northerly winds. These wind anomalies were favorable for coastal warming south of the equator through weakened upwelling and WES feedback, setting the stage for dramatic growth of the extreme coastal El Niño. In addition, WWEs from November 2016 and March 2017 excited strong downwelling Kelvin waves across the equatorial Pacific Ocean. In FM, the climatological SSTs of the southeastern tropical Pacific reach the annual maximum. The arrival of downwelling Kelvin waves during FM 2017 drove SSTs over CTSA above the convective threshold. Deep convection (Fig. 10, pink vectors), rare in the region, enhanced the northerly alongshore wind anomalies (Fig. 10, blue vectors), which in turn amplified the coastal warming. This coastal Bjerknes feedback is very important for the 2017 extreme coastal El Niño.

SSTs over CTSA do not usually exceed the convective threshold because coastal winds and Kelvin waves do not mutually cooperate: in JFMA, a sea level rise (drop) over CTSA is often associated with southerly (northerly) wind anomalies, and this positive correlation is apparent if the years of 1983, 1998, and 2017 are excluded (Fig. 1d). This can be explained as follows: during a moderate El Niño (La Niña), the EPID mode drives strong southerly (northerly) wind anomalies in the southeastern tropical Pacific (Fig. 8c, d) while the westerly (easterly) wind anomalies near the dateline (Fig. 1c) cause downwelling (upwelling) Kelvin waves, increasing (decreasing) sea level over CTSA (Fig. 1d). Thus the cooling (warming) effect of the southerly (northerly) anomalies oppose the warming (cooling) effect of downwelling (upwelling) Kelvin waves. As a result, deep convection hardly develops over CTSA. This explains why the extreme coastal El Niños are rare.

Nevertheless, in 2017, the alongshore northerly wind anomalies and downwelling equatorial Kelvin waves excited by the strong WWEs reinforced each other, driving SSTs off Peru and Ecuador above the threshold and activating the positive coastal Bjerknes feedback. We showed that initialized coupled models successfully predicted the 2017 event of extreme coastal rainstorms. Our study suggests that the successful prediction is enabled by the activation of the coastal Bjerknes feedback.

In a warming climate, the frequency of extreme coastal El Niño increases in most CMIP5 models and the MME mean (Supplementary Figure 2a). In addition, most climate models project an enhanced SST increase (Supplementary Figure 2b) in the southeastern Pacific compared to the tropical mean warming^25–27^. This reduces the barrier to deep convection off Peru and Ecuador and hence increases the frequency of extreme coastal El Niño (Supplementary Figure 2c, with an intermodel correlation *r* = 0.53). Supplementary Figure 2d further indicates that the frequency changes of extreme coastal El Niño are independent of the future basin-scale ENSO changes. Increases in extreme coastal El Niño occurrence have profound socio-economic consequences as in FMA 2017.

## Methods

### Extreme coastal El Niño

Both Niño 1 + 2 and Niño 3.4 SST indices are used to identify extreme coastal El Niño. An extreme coastal El Niño is defined as an event during which Niño 1 + 2 SSTs exceed the convective threshold while Niño 3.4 anomalies are negative. We define an extreme coastal El Niño as one with dramatic increase in rainfall off Peru and Ecuador but without basin-scale warming. The 2017 case fits this definition (Fig. 1b). The extreme coastal El Niño occurs in FM when SSTs off Peru reach the annual maximum and are close to the convective threshold.

### Observational data

Daily SST data were obtained from Operational Sea Surface Temperature and Sea Ice Analysis (OSTIA; ftp://podaac.jpl.nasa.gov/allData/ghrsst/data/L4/GLOB/UKMO/OSTIA/) on a 5 km × 5 km grid^28^. The OSTIA uses satellite data provided by the Group for High Resolution Sea Surface Temperature (GHRSST) project, together with in situ observations. We used the Air-Sea Fluxes for the Global Tropical Oceans (TropFlux) project (http://www.incois.gov.in/tropflux/) on a 1°× 1° grid^29^, monthly and daily wind velocity derived from the Cross-Calibrated Multi-Platform Version 2.0 (CCMP V2.0) on a 0.25° × 0.25° grid^30^, daily SLA from the Copernicus Marine and Environment Monitoring Service (CMEMS; http://www.marine.copernicus.eu) on a 0.25° ×  0.25° grid, monthly ocean temperature, currents, and sea surface height from the NCEP Global Ocean Data Assimilation System (GODAS; https://www.esrl.noaa.gov/psd/data/gridded/data.godas.html) on a 1° longitidue × (1/3)° latitude grid, and precipitation from the Tropical Rainfall Measuring Mission (TRMM; https://disc.gsfc.nasa.gov/datacollection/TRMM_3B43_7.html) on a 0.25° × 0.25° grid. Monthly SST and regional SST indices for the equatorial Pacific, including the Niño 3.4 (5°S–5 °N, 170 °W–120 °W) and Niño 1+2 (0–10 °S, 90 °W–80 °W) regions, are from the NOAA Optimum Interpolation Sea Surface Temperature version 2 dataset (OISST; https://www.esrl.noaa.gov/psd/data/gridded/data.noaa.oisst.v2.html)^31^. The wind anomalies in Niño1 + 2 and Niño4 (5 °N–5 °S, 160 °E–150 °W) were obtained from the Interim European Centre for Medium-Range Weather Forecasts (ECMWF) ReAnalysis (ERA-Interim) products (http://apps.ecmwf.int/datasets/data/interim-full-daily/levtype=sfc/) for 1982 to 2017 on a 0.5° × 0.5° grid^32^. The Real-time Multivariate MJO indices (RMM1 and RMM2) of Wheeler and Hendon^33^ were used to track the MJO (http://www.bom.gov.au/climate/mjo/graphics/rmm.74toRealtime.txt).

### Model experiments

OGCM: We performed experiments with MITgcm^34^ to examine the SST response to the local and remote forcing. The model is based on a LLC90 grid^35^ with 1° zonal resolution and meridional resolution varying from 0.4° at the equator to 1° at mid-latitudes. There are 50 vertical levels, with layer thickness gradually increasing from 5 m near the surface to about 450 m in the deep ocean. The surface forcing fields include six-hourly zonal and meridional surface wind stress, wind speed, specific humidity, downward longwave and shortwave, and precipitation from ERA-Interim. Wind stress and wind speed are imposed separately in the model because they affect ocean temperature through different processes. Wind stress drives ocean dynamics (advection and upwelling) and mixing while wind speed affects ocean temperature mainly through surface turbulent heat flux (latent plus sensible heat flux). In our model, turbulent heat fluxes are calculated online with wind speed, air temperature, specific humidity, and modeled SST using bulk formula. The model restarted from an initial state obtained from Estimating the Circulation and Climate of the Ocean Version 4 Release 3 (refs. ^35,36^) (ECCO v4r3), and is integrated forward from 1 January 2007 to 31 December 2017.

To isolate various effects on the coastal warming, we have performed four experiments (Supplementary Table 2). The control run (OCTL) is forced with full observed forcing fields for 2007–2017. In the no coastal wind run (NoCoastW), climatological wind stress is imposed in the CTSA during November 2016–May 2017, while other forcing is retained. In the coastal wind run (CoastW), climatological wind stress is imposed outside the CTSA, whereas other observed forcing is retained. In the no wind run (NoW), we keep the wind stress everywhere fixed to the monthly climatology during November 2016–May 2017 to assess the effect of thermal forcing on SST. The difference solutions, NoCoastW-NoW and CoastW-NoW, isolate the effect of remote and local wind stress forcing, respectively.

AGCM: The CAM5.3 used in this study is a recent global atmosphere model of the National Center for Atmospheric Research (NCAR), and the atmospheric component of the Community Earth System Model version 1.2.2 (ref. ^37^) (CESM1.2.2). The model resolution is 0.9° latitude × 1.25° longitude (“f09_f09”) with 30 sigma levels in the vertical.

In order to isolate the atmospheric response to the 2017 coastal El Niño, We conduct three experiments. In the control run (ACTL), the model is forced by the monthly climatology of SST and sea ice during 1980–2017 from the Hadley Centre Global Sea Ice and Sea Surface Temperature (HadISST). In the A2017 run, we impose observed monthly SSTAs in 2017 over the world ocean. In the coastal run (ACoast2017), we only impose 2017 SSTAs in the southeastern Pacific region (90 °W–75 °W, 20 °S–0°). Each run lasts for 11 years and the results of the last 10 years are analyzed.

*CGCM*: We evaluate the seasonal forecast from the NMME (https://iridl.ldeo.columbia.edu/SOURCES/.Models/.NMME/)^38^. Seven models from the NMME are used: CMC1, CMC2, CCSM4, GFDL CM2.1, GFDL CM2.5 FLOR A06, GFDL CM2.5 FLOR B01, and NCEP CFSv2. All the model data are provided with 1° × 1° resolution.

To evaluate how the extreme coastal El Niño changes under global warming, we used the output of 22 models during 1900–2100 from the Coupled Model Intercomparison Project phase 5 (ref. ^39^) (CMIP5) organized by the Program for Climate Model Diagnosis and Intercomparison. Relative SST is defined as the local SST deviation from the tropical mean over 20 °S–20 °N^40^.

### A mixed layer heat budget

The heat budget in the mixed layer can be expressed as

1 $$\frac{{\partial T}}{{\partial t}} = \frac{{Q_{{\mathrm {net}}} - Q_{{\mathrm {pen}}}}}{{\rho C_ph}} - v \cdot \nabla T + {\mathrm {subsurface}},$$

where *h* is the mixed layer depth, *T* and *v* are temperature and horizontal currents averaged over the mixed layer, *ρ* and *C*_p_ are density (1027 kg m^−^^3^) and specific heat capacity of seawater (4007 J kg^−1^ K^−1^). The first term on the rhs represents the effect of surface heat flux. *Q*_net_ represents the net surface heat flux and *Q*_pen_ is the shortwave radiation transmitted through the bottom of  mixed layer depth parameterized as^41^

2 $$Q_{{\mathrm {pen}}} = Q_{{\mathrm {short}}}\left( {0.58e^{ - h/0.35} + 0.42e^{ - h/23}} \right),$$

where *Q*_short_ is the shortwave heat flux. The second term of Eq. (1) represents the effect of horizontal advection. The third term is the subsurface term, including vertical entrainment and diffusion. Here we estimate the subsurface term as the residual of Eq. (1).

### Code availability

The code and scripts used to analyze the data and to generate the plots in this paper are available from the corresponding author on request.

## Supplementary information

Supplementary Information

## Data Availability

All data used in this study are available online or from the corresponding author on request.

## References

[1] Johnson NC, Xie SP (2010). Changes in the sea surface temperature threshold for tropical convection. Nat. Geosci..

[2] Garreaud RD (2018). A plausible atmospheric trigger for the 2017 coastal El Niño. Int. J. Climatol..

[3] Bjerknes J (1969). Atmospheric teleconnections from the equatorial Pacific. Mon. Weather Rev..

[4] Wyrtki K (1975). El Niño—the dynamic response of the equatorial Pacific Ocean to atmospheric forcing. J. Phys. Oceanogr..

[5] Rasmusson EM, Carpenter TH (1982). Variations in tropical sea surface temperature and surface wind fields associated with the Southern Oscillation/El Niño. Mon. Weather Rev..

[6] Deser C, Wallace JM (1990). Large-scale atmospheric circulation features of warm and cold episodes in the Tropical Pacific. J. Clim..

[7] Ashok K, Behera SK, Rao SA, Weng H, Yamagata T (2007). El Niño Modoki and its possible teleconnection. J. Geophys. Res..

[8] Kao HY, Yu JY (2009). Contrasting eastern-Pacific and central-Pacific types of ENSO. J. Clim..

[9] Kug JS, Jin FF, An SI (2009). Two types of El Niño events: cold tongue El Niño and warm pool El Niño. J. Clim..

[10] Takahashi K, Montecinos A, Goubanova K, Dewitte B (2010). ENSO regimes: reinterpreting the canonical and Modoki El Niño. Geophys. Res. Lett..

[11] Takahashi, K. & Martínez, A. G. The very strong coastal El Niño in 1925 in the far-eastern Pacific. *Clim. Dyn*. 1–27 (2018).

[12] McPhaden MJ (1990). Genesis and evolution of the 1997-98 El Niño. Science.

[13] Okumura YM, Deser C (2010). Asymmetry in the duration of El Niño and La Niña. J. Clim..

[14] Vecchi GA, Harrison DE (2000). Tropical Pacific sea surface temperature anomalies, El Niño, and equatorial westerly wind events. J. Clim..

[15] Yu JY, Kao HY, Lee T (2010). Subtropics-related interannual sea surface temperature variability in the central equatorial Pacific. J. Clim..

[16] Ma J, Xie SP, Xu H (2017). Inter-member variability of the summer northwest Pacific subtropical anticyclone in the ensemble forecast. J. Clim..

[17] Hu, Z. Z., Huang, B., Zhu, J., Kumar, A. & McPhaden, M. J. On the variety of coastal El Niño events. *Climate Dyn*. 1–16 (2018).

[18] Xie SP (1994). & Philander S. G. H. A coupled ocean-atmosphere model of relevance to the ITCZ in the eastern Pacific. Tellus A.

[19] Xie SP (2018). Eastern Pacific ITCZ dipole and ENSO diversity. J. Clim..

[20] Comité Multisectorial Encargado del Estudio Nacional del Fenómeno El Niño (ENFEN). Informe Técnico Enfen. Año 3, N^o^ 03, marzo de 2017. 65 (2017)

[21] Takahashi K (2018). The 2017 coastal El Niño. Bull. Am. Meteor. Soc..

[22] Vecchi, G. A., Wittenberg, A. T. & Rosati, A. Reassessing the role of stochastic forcing in the 1997–1998 El Niño. *Geophys. Res. Lett*. **33** (2006).

[23] Chen D (2015). Strong influence of westerly wind bursts on El Niño diversity. Nat. Geosci..

[24] Hu, S. & Fedorov, A. V. The extreme El Niño of 2015–2016: the role of westerly and easterly wind bursts, and preconditioning by the failed 2014 event. *Climate Dyn*. 1–19 (2017).

[25] Xie SP (2010). Global warming pattern formation: sea surface temperature and rainfall. J. Clim..

[26] Long SM, Xie SP, Liu W (2016). Uncertainty in tropical rainfall projections: atmospheric circulation effect and the ocean coupling. J. Clim..

[27] Power S, Delage F, Chung C, Kociuba G, Keay K (2013). Robust twenty-first-century projections of El Niño and related precipitation variability. Nature.

[28] Donlon CJ (2012). The operational sea surface temperature and sea ice analysis (OSTIA) system. Remote Sens. Environ..

[29] Kumar BP, Vialard J, Lengaigne M, Murty VSN, McPhaden MJ (2012). TropFlux: air-sea fluxes for the global tropical oceans-description and evaluation. Clim. Dyn..

[30] Atlas R (2011). A cross-calibrated, multiplatform ocean surface wind velocity product for meteorological and oceanographic applications. Bull. Am. Meteor. Soc..

[31] Reynolds RW, Rayner NA, Smith TM, Stokes DC, Wang WQ (2002). An improved in situ and satellite SST analysis for climate. J. Clim..

[32] Dee DP (2011). The ERA-Interim reanalysis: configuration and performance of the data assimilation system. Quart. J. R. Meteor. Soc..

[33] Wheeler MC, Hendon HH (2004). An all-season real-time multivariate MJO index: development of an index for monitoring and prediction. Mon. Weather Rev..

[34] Marshall J, Adcroft A, Hill C, Perelman L, Heisey C (1997). A finite volume, incompressible Navier-Stokes model for studies of the ocean on parallel computers. J. Geophys. Res..

[35] Forget GAEL (2015). ECCO version 4: an integrated framework for non-linear inverse modeling and global ocean state estimation. Geosci. Model Dev..

[36] Fukumori, I. et al. ECCO Version 4 Release 3. *NASA-JPL*. Pasadena, CA (2017).

[37] Neale, R. B. et al. Description of the NCAR Community Atmosphere Model (CAM 5.0). *Tech. Report.* NCAR, Boulder, Colo (2012).

[38] Kirtman BP (2014). The North American multimodel ensemble: phase-1 seasonal-to-interannual prediction; phase-2 toward developing intraseasonal prediction. Bull. Am. Meteor. Soc..

[39] Taylor KE, Stouffer RJ, Meehl GA (2012). An overview of CMIP5 and the experiment design. Bull. Am. Meteor. Soc..

[40] Zheng XT, Xie SP, Lv LH, Zhou ZQ (2016). Intermodel uncertainty in ENSO amplitude change tied to Pacific Ocean warming pattern. J. Clim..

[41] Paulson CA, Simpson JJ (1977). Irradiance measurements in the upper ocean. J. Phys. Oceanogr..

