# Supplementary material for: Coupled ocean-atmosphere dynamics of the 2017 extreme coastal El Niño
Source: Nat Commun. 2019 Jan 17;10:298. doi: 10.1038/s41467-018-08258-8 (PMC6336809; doi:10.1038/s41467-018-08258-8)
Supplement: Supplementary file 1 — Supplementary Information [file 41467_2018_8258_MOESM1_ESM.pdf]

Coupled ocean-atmosphere dynamics of the 2017 extreme coastal El Niño

Qihua Peng[1,2,3], Shang-Ping Xie[2,4*], Dongxiao Wang[1*], Xiao-tong Zheng[4] and Hong Zhang[5]

[1]State Key Laboratory of Tropical Oceanography, South China Sea Institute of Oceanology, Chinese Academy of Sciences, Guangzhou, China.

[2]Scripps Institution of Oceanography, University of California San Diego, La Jolla, California 92093, USA.

[3]University of Chinese Academy of Sciences, Beijing, China.

[4]Physical Oceanography Laboratory, Ocean University of China, and Qingdao National Laboratory for Marine Science and Technology, Qingdao 266100, China.

[5] Joint Institute for Regional Earth System Science and Engineering, University of California, Los Angeles, CA, USA.

Supplementary Table 1. In-situ observations of SSTAs and sea level anomalies from stations along the coast of Peru[1]

Station	SSTAs (^{o}C)						SLA (cm)					
	Nov	Dec	Jan	Feb	Mar	Apr	Nov	Dec	Jan	Feb	Mar	Apr
Talara (4.5^{o}S, 81°W)	-2.3	-1.3	1.0	3.0	4.3	0.0	-1	0	4	8	14	10
Paita (5°S, 81°W)	-0.1	-0.1	2.4	4.6	5.7	2.3	1	1	6	11	15	10
Isla Lobos de Afuera (7°S, 80°W)	-0.5	-1.0	0.7	4.2	5.8	2.1	-4	-3	3	7	11	8
Chimbote (9°S, 78 ^{o}W)	0.6	-0.2	-0.2	2.6	4.4	1.4	-2	-2	1	7	11	9
San Juan (12°S, 77 ^{o}W)	0.4	0.0	0.5	0.6	0.9	0.6	-5	3	5	3	7	6

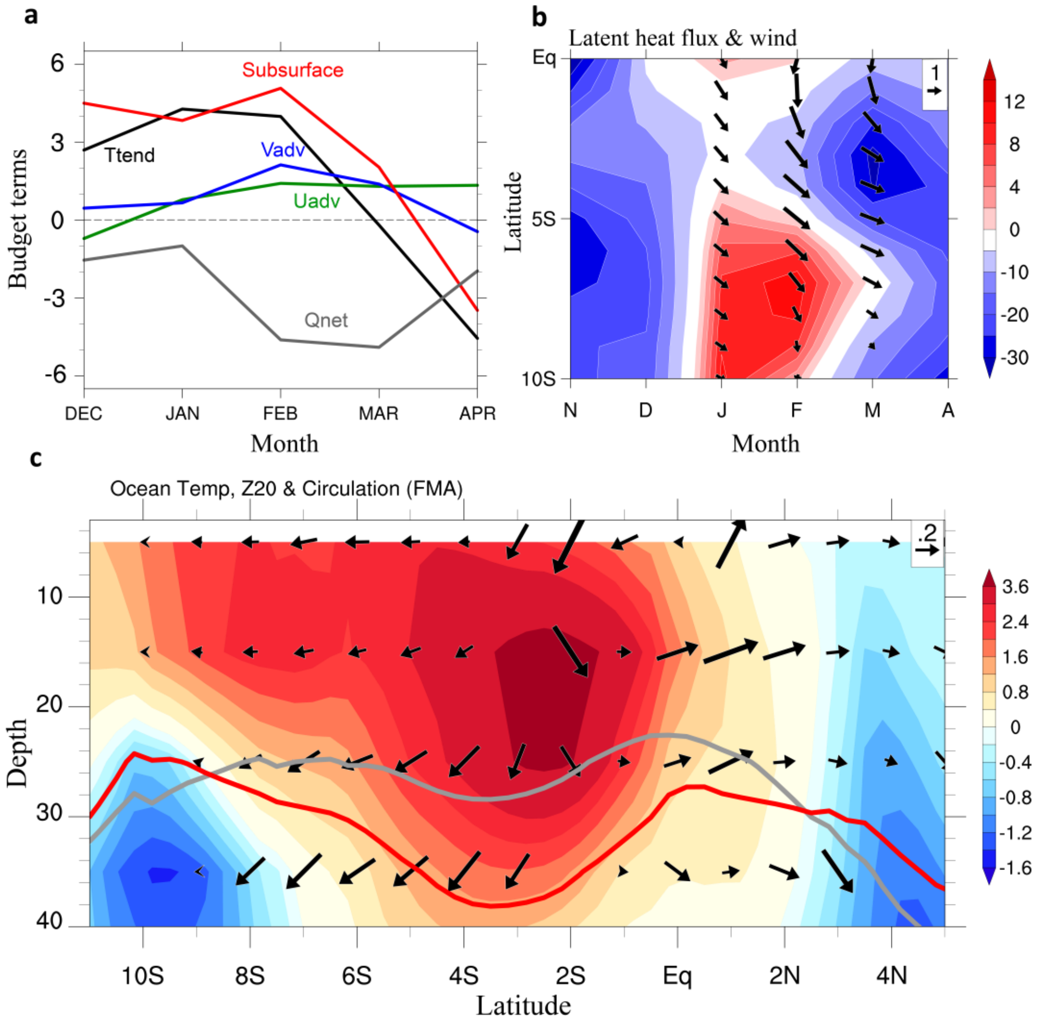

Supplementary Figure 1. Heat budget of the 2017 extreme coastal El Niño. (a) Ocean mixed layer budget (10^{-7} °C s^{-1}) averaged in 80°W-85°W, 10°S-0: mixed layer temperature tendency (Ttend, black line), Net heat flux term (Qnet, grey line), zonal advection term (Uadv, green line), meridional advection term (Vadv, blue line), and subsurface term (red line). (b) Latitude-time evolution of latent heat flux anomalies (W m^{-2}; color shading) and surface wind anomalies (m s^{-1}; vectors). (c) Cross-equatorial transect of anomalous vertical circulation (arrows, m s^{-1} in meridional direction and 10^{-5}m s^{-1} in vertical direction) and ocean temperature (°C; color shading) in FMA zonally averaged in 80°W-85°W, along with the 20°C isotherm depth for FMA climatological value (grey line) and 2017 FMA (red line).

Supplementary Table 2. Description of the MITgcm experiments

Experiment	Forcing	Description
OCTL	6-hourly full forcing	Complete
NoCoastW	Same as OCTL but for climatological wind stress in CTSA from November 2016-May 2017	Remote forcing effect + Local thermal forcing
CoastW	Same as OCTL but for climatological wind stress outside CTSA from November 2016-May 2017	Local wind effect + Local thermal forcing
NoW	Same as OCTL but for climatological wind stress all over the world ocean from November 2016-May 2017	Local thermal forcing

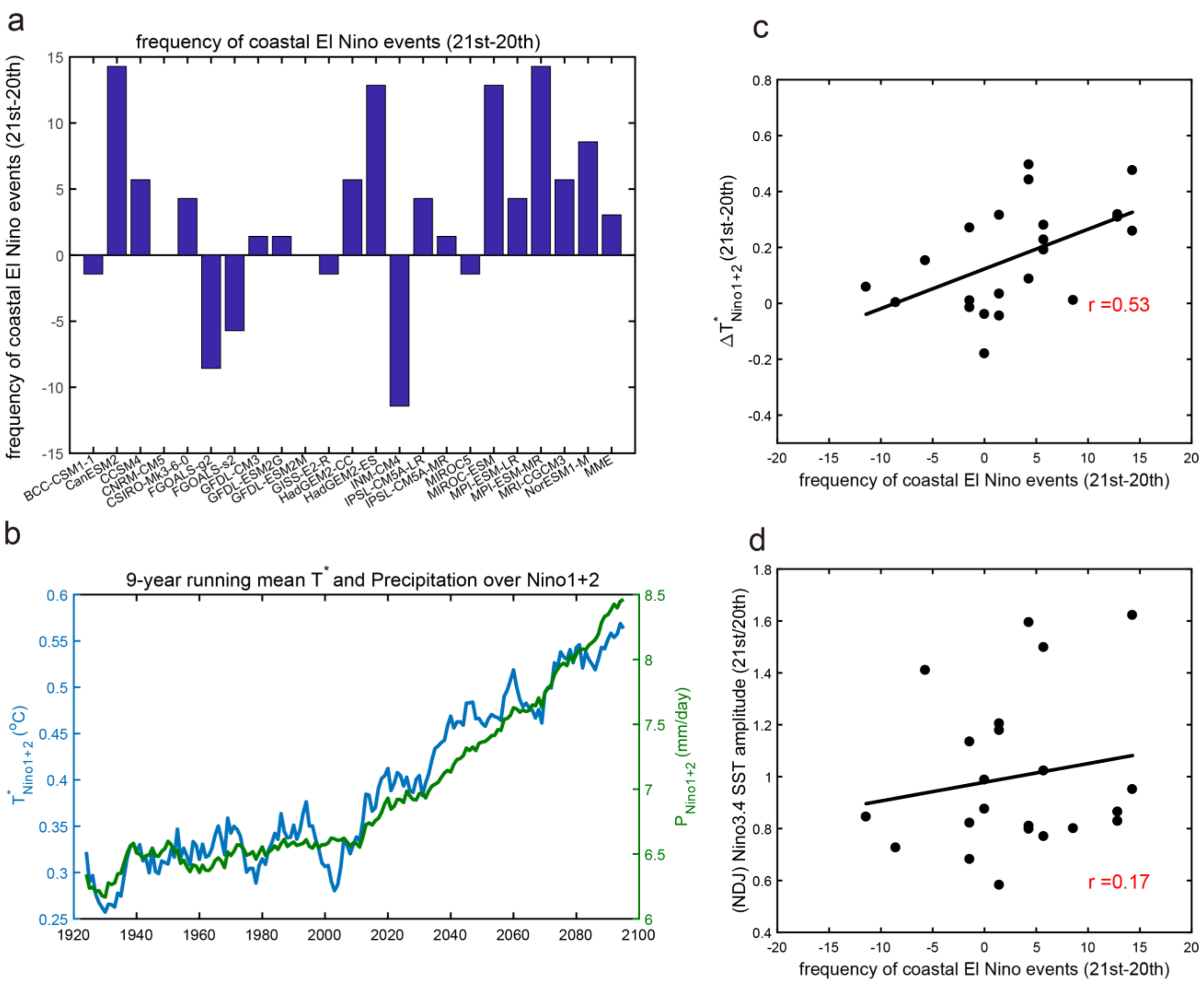

Supplementary Figure 2. Changes of extreme coastal El Niño under global warming. (a) The 21st to 20th century difference in frequency (per century) of extreme coastal El Niño. (b) The Coupled Model Intercomparison Project phase 5 (CMIP5) Inter-model ensemble 9 year running mean relative SSTs (T*, °C; blue line) and rainfall (mm day^{-1}; green line) over Niño 1+2 region. (c) Inter-model scatterplots of the frequency of extreme coastal El Niño with the 21st to 20th century difference of Niño 1+2 relative SSTs. The correlation is significant at the 95% confidence level based on t-test. (d) Same as (c) but with the 21st to 20th ratio of Niño 3.4 SST amplitude.

Supplementary References

1. Comité Multisectorial Encargado del Estudio Nacional del Fenómeno El Niño (ENFEN). 2017. *Informe Técnico Enfen. Año 3, N° 03, Abril*, 65 (2017).
